# A rapid progression from classical mantle cell lymphoma to a blastoid variant

**DOI:** 10.1016/j.lrr.2024.100484

**Published:** 2024-11-05

**Authors:** Radu Chiriac, Marie Donzel, Lucile Baseggio

**Affiliations:** a Hospices Civils de Lyon, Centre Hospitalier Lyon Sud, Laboratoire d'hématologie biologique, Pierre-Bénite, France; b Hospices Civils de Lyon, Centre Hospitalier Lyon Sud, Service d'anatomie pathologique, Pierre-Bénite, France

**Keywords:** B-cell lymphoma, CCND1, Bendamustine

## Abstract

This case report presents an 82-year-old male initially diagnosed with classical mantle cell lymphoma (MCL) that progressed to the aggressive blastoid variant. The patient was initially treated with oral chemotherapy (PEP-C), followed by ibrutinib, but experienced disease progression with central nervous system (CNS) involvement and blastoid morphology. Despite subsequent intensive treatment, including high-dose cytarabine (Cytarabine), rituximab, and intrathecal methotrexate (Methotrexate), the patient's disease continued to advance, resulting in death. This case underscores the aggressive nature of blastoid MCL, its poor prognosis despite current therapeutic strategies, and highlights the need for individualized treatment approaches and CNS prophylaxis.

## 1 Introduction

Mantle cell lymphoma (MCL) is a well-established type of mature B-cell lymphoma characterized by a monomorphic proliferation of small lymphoid cells that carry t(11;14)(q13;q32) (IGH*::CCND1* rearrangement) resulting in cyclin D1 overexpression [1].

Blastoid MCL is highly aggressive with a poor prognosis, characterized by specific cytomorphological features and a high Ki-67 labeling index, which offers better prognostic information than cytology subtypes [2]. In clinical cohorts, these subsets occur in approximately 10 % of cases [3].

Standard chemotherapy regimens for MCL, such as bendamustine, rarely achieve prolonged remissions at dosages used for classical variants [4]. Therefore, high-dose cytarabine (Cytarabine) regimens with intensive consolidation are generally recommended due to the more aggressive clinical course of these patients. However, even with intensified treatments, long-term outcomes remain poor [5].

In this report, we present a case initially diagnosed as classical MCL that progressed to a more aggressive form, the blastoid variant.

## 2 Case presentation

An 82-year-old man was diagnosed with MCL four years ago. He presented with night sweats, weight loss (6 kg in one month), and right cervical adenopathy; no splenomegaly was noted. Fluorodeoxyglucose positron emission tomography-computed tomography (FDG PET/CT) showed increased FDG uptake in the cervical, axillary, and retroperitoneal areas.

**Fig. 1:**
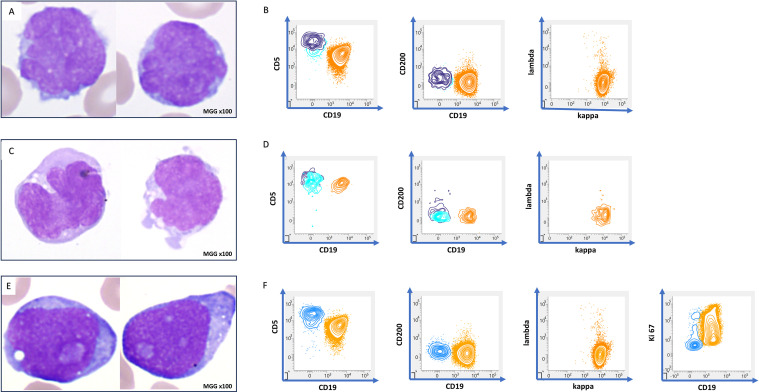
Panels A, C, E (MGG stain, 100x objective) show various morphologic aspects, from classical (A) to blastoid (E) forms. Panels B, D, and F display flow cytometry phenotype, with the orange plot indicating CD19-positive lymphoma B-cells.

**Fig. 2:**
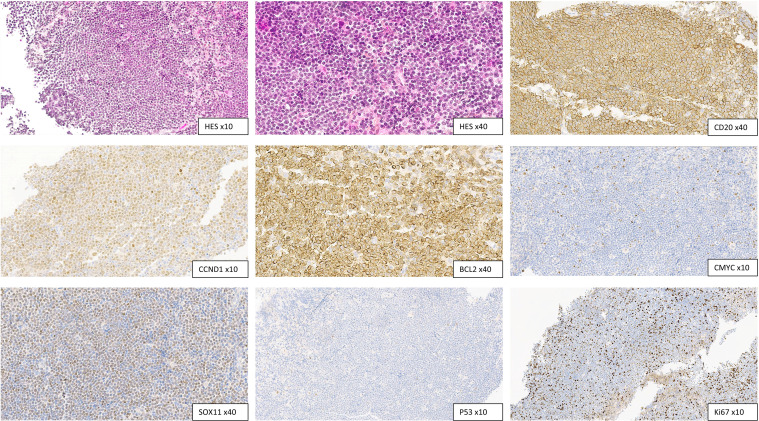
Initial lymph node biopsy showing a classical phenotype: CD20+, cyclin D1+, BCL2+, C-Myc-, SOX11+, p53-, and Ki-67 index estimated at15 %.

Initial blood work revealed white blood cells (WBC) at 6.5 × 10^9^/L, hemoglobin at 120 g/L, platelets at 120×10^9^/L, and lymphocytes at 2.5 × 10^9^/L. Blood smears revealed medium-sized lymphocytes characterized by irregular nuclei with mature clumped chromatin, slight nucleoli, and scant pale cytoplasm (Fig. 1, Panel A). Peripheral blood (PB) flow cytometry (FCM) detected a monoclonal kappa CD19+/CD20+ *B*-cell population comprising 50 % of total lymphocytes. This population expressed bright CD5 and was negative for CD23 and CD200 (Fig. 1, Panel B, orange plot). The immunophenotypic profile and cytomorphology suggested a minimal dissemination of MCL in PB. A subsequent bone marrow examination showed lymphoma involvement, with 20 % monoclonal B cells detected by FCM. In addition, a lymph node biopsy favored a diagnosis of classical MCL, with a typical phenotype: CD5+, cyclin D1+, SOX11+, BCL2+, BCL6-, CD10-, and CD23-. There was no CMYC- or p53 overexpression and Ki67 index was estimated at 15 % (Fig. 2). Bone marrow chromosomal analysis demonstrated complex karyotypic abnormalities, including t(11;14)(q13;q32) as part of the following: 44,X,-Y,del(1)(q31), del(6)(q13), 7,i(8)(q10), del(10)(p11), del(11)(q14q23), t(11;14)(q13;q32), dup(12)(q13q23), der(13)add(13)(p11)del(13)(q13q21), del(16)(q21)[9]/44, idem, add(2)(p25), t(5;6)(p11;q11), -i(8)(q10)[9]/44, idem, add(2)(p25), t(5;6), -i(8)(q10). Fluorescence in situ hybridization was positive for the IGH::*CCND1* fusion in metaphase cells, thereby confirming the diagnosis of MCL.

Due to refusal of intravenous treatment, the oral combination regimen of prednisone 20 mg, etoposide (Etoposide) 50 mg, procarbazine 50 mg, cyclophosphamide 50 mg (PEP-C) was employed. After 15 months, the patient returned for consultation due to the reappearance of cervical, axillary, mediastino-hilar, retroperitoneal, and mesenteric adenopathies. A change to ibrutinib (560 mg orally each day) was initiated, planned for two years, with a good initial response. However, after three months, due to a new flare-up of lymphoma, characterized by autoimmune hemolysis under ibrutinib and an increase in cervical adenopathies, a change in treatment to aracytine and rituximab was motivated. The new regimen included dexamethasone 40 mg on days 1–4, rituximab 500 mg on day 1, and cytarabine (Cytarabine) 3000 mg/m² twice daily on days 2–3, in 21-day cycles.

One year later, the patient was hospitalized in the intensive care unit due to rapid health deterioration, binocular diplopia, and bilateral cervical adenopathies with edema. Elevated lactate dehydrogenase was noted at 1500 U/L (normal range 150–300 U/L). No circulating lymphomatous cells were observed. A lumbar puncture was performed, and the cytospin preparation of the cerebrospinal fluid (CSF) revealed lymphomatous infiltration (Fig. 1, Panel C), confirmed by FCM (Fig. 1, Panel D, orange plot). Central nervous system (CNS) involvement of the MCL was finally confirmed. Intrathecal high-dose methotrexate (Methotrexate) was administered at 2 g/m², and R-Bendamustine was initiated. The bendamustine dosing was 70 mg/m² on days 1 and 2 per cycle, every 28 days for up to six cycles, with the rituximab dose being 375 mg/m² for the first course and 500 mg/m² for all subsequent courses. This treatment regimen resulted in a regression of symptoms.

Six months later, the patient presented with a recurrence of compressive cervical adenopathy. Blood work showed lymphocytosis (97×10^9^/L), with 90 % of blast-like cells exhibiting medium-sized, round nuclei, immature chromatin, prominent central nucleoli and basophilic cytoplasm, corresponding to a blastoid morphology (Fig. 1, Panel E). FCM studies confirmed the presence of circulating MCL with a similar phenotype to the initial diagnosis, but now with more proliferative characteristics (Ki-67 index >90 %) (Fig. 1, Panel F, orange plot). No lymph nodes were accessible due to the patient's fragile condition. In this context, supportive care was implemented, including palliative care and the introduction of corticosteroids at a dose of 70 mg/day. The patient died one week after the diagnosis of this blastoid progression.

## 3 Discussion

Blastoid morphology may be the initial presentation of MCL at diagnosis [2,3]. However, in some cases, the morphology may evolve from classical to blastoid during disease progression [6]. Genomic alterations observed in blastoid variants might precede this transformation [7]. Although blastoid MCL presents similarly to classical MCL, systematic comparisons are limited. This case highlights the clinical challenges and poor prognosis of MCL, particularly the aggressive transition from classical to blastoid variant. Initially, the patient exhibited minimal blood involvement characterized by classic morphology and was treated with appropriate therapy. Despite this, the disease progressed, manifesting as CNS involvement and lymphocytosis with blastoid morphology. FCM revealed a high rate of proliferation. The patient's initial treatment with oral combination chemotherapy (PEP-C) was followed by a period of stability. However, the subsequent emergence of autoimmune hemolysis and worsening adenopathies led to a switch to ibrutinib. Despite an initial response, the patient's condition deteriorated, prompting a change to a more intensive regimen of aracytine and rituximab. This regimen led to some symptom regression but was followed by a recurrence of compressive cervical adenopathy and blastoid cell morphology. Despite the intensified treatment approach including high-dose cytarabine (Cytarabine), the long-term prognosis remained unfavorable, highlighting the limitations of current therapies for such aggressive forms of MCL.

Furthermore, the development of CNS involvement, confirmed by CSF analysis, adds another layer of complexity to management. In various series, blastoid cytology predisposes for CNS involvement [8]. In a retrospective survey by the European MCL Network, 57 of 1396 patients (4.1 %) had CNS involvement, including 0.9 % at diagnosis [9]. CNS prophylaxis may be considered for blastoid MCL, but the efficacy of intrathecal or systemic treatments with methotrexate (Methotrexate) or high-dose cytarabine (Cytarabine) remains inconclusive [10]. The patient's final treatment regimen, involving intrathecal high-dose methotrexate (Methotrexate) and R-Bendamustine, resulted in some regression of symptoms but did not prevent the subsequent relapse.

The challenges faced in managing this aggressive lymphoma variant highlight the necessity for tailored treatment approaches and continued exploration of novel therapies to improve outcomes. In terms of diagnosis and treatment, blastoid MCL presents significant challenges. Typically, cases are characterized by high cell proliferation and numerous genetic alterations. Considering the aggressive clinical course typically observed, adopting a watch-and-wait strategy is generally not recommended, even in cases characterized by low tumor burden. Despite an optimized therapeutic approach, blastoid MCL frequently leads to poor long-term outcomes. Consequently, an allogeneic approach may be considered early in the disease.

## 4 Conclusion

Although classic MCL typically responds well to new therapies with a favorable prognosis, this case illustrates that relapse and/or evolution to a more aggressive form, particularly the blastoid variant, can occur. Such progression is associated with a poor prognosis and necessitates tailored treatment and CNS prophylaxis.

## Data availability

Data sharing is not applicable to this article as no new data were created or analysed in this study.

## Funding statement

n/a.

## Ethics approval statement

n/a.

## Patient consent statement

Written informed consent was obtained from the patient's next of kin to publish this report in accordance with the journal's patient consent policy.

## Permission to reproduce material from other sources

The authors declare no use of third-party material in this study for which formal permission is required.

## Clinical trial registration

n/a.

## CRediT authorship contribution statement

**Radu Chiriac:** Conceptualization, Writing – original draft. **Marie Donzel:** Methodology, Visualization. **Lucile Baseggio:** Formal analysis, Visualization.

## Declaration of competing interest

The authors declare that they have no known competing financial interests or personal relationships that could have appeared to influence the work reported in this paper.

## References

[1] Jain P., Wang M. (2019). Mantle cell lymphoma: 2019 update on the diagnosis, pathogenesis, prognostication, and management. Am. J. Hematol..

[2] Hoster E., Rosenwald A., Berger F., Bernd H.W., Hartmann S., Loddenkemper C., Barth T.F., Brousse N., Pileri S., Rymkiewicz G., Kodet R., Stilgenbauer S., Forstpointner R., Thieblemont C., Hallek M., Coiffier B., Vehling-Kaiser U., Bouabdallah R., Kanz L., Pfreundschuh M., Schmidt C., Ribrag V., Hiddemann W., Unterhalt M., Kluin-Nelemans J.C., Hermine O., Dreyling M.H., Klapper W (2016). Prognostic value of Ki-67 index, cytology, and growth pattern in mantle-cell lymphoma: results from randomized trials of the European mantle cell lymphoma network. J. Clin. Oncol..

[3] Jain P., Wang M. (2020). Blastoid Mantle Cell Lymphoma. Hematol. Oncol. Clin. North Am..

[4] Visco C., Chiappella A., Nassi L., Patti C., Ferrero S., Barbero D., Evangelista A., Spina M., Molinari A., Rigacci L., Tani M., Rocco A.D., Pinotti G., Fabbri A., Zambello R., Finotto S., Gotti M., Carella A.M., Salvi F., Pileri S.A., Ladetto M., Ciccone G., Gaidano G., Ruggeri M., Martelli M., Vitolo U. (2017). Rituximab, bendamustine, and low-dose cytarabine as induction therapy in elderly patients with mantle cell lymphoma: a multicentre, phase 2 trial from Fondazione Italiana Linfomi. Lancet Haematol..

[5] Laurell A., Kolstad A., Jerkeman M., Räty R., Geisler C.H. (2014). High dose cytarabine with rituximab is not enough in first-line treatment of mantle cell lymphoma with high proliferation: early closure of the Nordic lymphoma group mantle cell lymphoma 5 trial. Leuk. Lymphoma.

[6] Vogt N., Klapper W. (2013). Variability in morphology and cell proliferation in sequential biopsies of mantle cell lymphoma at diagnosis and relapse: clinical correlation and insights into disease progression. Histopathology.

[7] Greiner T.C., Moynihan M.J., Chan W.C., Lytle D.M., Pedersen A., Anderson J.R., Weisenburger D.D. (1996). p53 mutations in mantle cell lymphoma are associated with variant cytology and predict a poor prognosis. Blood.

[8] Ferrer A., Bosch F., Villamor N., Rozman M., Graus F., Gutiérrez G., Mercadal S., Campo E., Rozman C., López-Guillermo A., Montserrat E. (2008). Central nervous system involvement in mantle cell lymphoma. Ann. Oncol..

[9] Cheah C.Y., George A., Giné E., Chiappella A., Kluin-Nelemans H.C., Jurczak W., Krawczyk K., Mocikova H., Klener P., Salek D., Walewski J., Szymczyk M., Smolej L., Auer R.L., Ritchie D.S., Arcaini L., Williams M.E., Dreyling M., Seymour J.F., European Mantle Cell Lymphoma Network (2013). Central nervous system involvement in mantle cell lymphoma: clinical features, prognostic factors and outcomes from the European mantle cell lymphoma network. Ann. Oncol..

[10] Dreyling M., Campo E., Hermine O., Jerkeman M., Le Gouill S., Rule S., Shpilberg O., Walewski J., Ladetto M., ESMO Guidelines Committee (2017). Newly diagnosed and relapsed mantle cell lymphoma: ESMO clinical practice guidelines for diagnosis, treatment and follow-up. Ann. Oncol..

